# The angiotensin converting enzyme inhibitor, captopril, prevents the hyperactivity and impulsivity of neurokinin-1 receptor gene ‘knockout’ mice: Sex differences and implications for the treatment of attention deficit hyperactivity disorder

**DOI:** 10.1016/j.euroneuro.2015.01.013

**Published:** 2015-04

**Authors:** Ashley J. Porter, Katharine Pillidge, Ewelina M. Grabowska, S. Clare Stanford

**Affiliations:** Department of Neuroscience, Physiology & Pharmacology, University College London, Gower St, London WC1E 6BT, UK

**Keywords:** ADHD, Angiotensin receptor, Captopril, Hyperactivity, Impulsivity, Neurokinin-1 receptor

## Abstract

Mice lacking functional neurokinin-1 receptors (NK1R−/−) display behavioural abnormalities resembling attention deficit hyperactivity disorder (ADHD): locomotor hyperactivity, impulsivity and inattentiveness. The preferred ligand for NK1R, substance P, is metabolised by angiotensin converting enzyme (ACE), which forms part of the brain renin angiotensin system (BRAS). In view of evidence that the BRAS modulates locomotor activity and cognitive performance, we tested the effects of drugs that target the BRAS on these behaviours in NK1R−/− and wildtype mice. We first tested the effects of the ACE inhibitor, captopril, on locomotor activity. Because there are well-established sex differences in both ADHD and ACE activity, we compared the effects of captopril in both male and female mice. Locomotor hyperactivity was evident in male NK1R−/− mice, only, and this was abolished by treatment with captopril. By contrast, male wildtypes and females of both genotypes were unaffected by ACE inhibition. We then investigated the effects of angiotensin AT_1_ (losartan) and AT_2_ (PD 123319) receptor antagonists on the locomotor activity of male NK1R−/− and wildtype mice. Both antagonists increased the locomotor activity of NK1R−/− mice, but neither affected the wildtypes. Finally, we tested the effects of captopril on the performance of male NK1R−/− and wildtype mice in the 5-choice serial reaction-time task (5-CSRTT) and found that ACE inhibition prevented the impulsivity of NK1R−/− mice. These results indicate that certain behaviours, disrupted in ADHD, are influenced by an interaction between the BRAS and NK1R, and suggest that ACE inhibitors could provide a novel treatment for this disorder.

## 1 Introduction

Male mice with functional ablation of the *Nk1r* gene, which encodes the substance P-preferring NK1 receptor (NK1R−/−), express locomotor *hyperactivity* in several experimental contexts (Fisher et al., 2007; Herpfer et al., 2005; Yan et al., 2010). In the 5-choice serial reaction-time task (5-CSRTT), a procedure that is used to evaluate cognitive performance, NK1R−/− mice also express more omissions (*inattentiveness*) and more premature responses (*motor impulsivity*) compared with their wildtypes (Dudley et al., 2013; Yan et al., 2011). Hyperactivity, inattentiveness and impulsivity are diagnostic criteria for attention deficit hyperactivity disorder (ADHD). On this basis, and supported by evidence from human genetic studies (Sharp et al., 2014), we have proposed that polymorphism(s) of the *TACR1* gene (the human equivalent of the mouse *Nk1r* gene) could be associated with increased risk of developing ADHD.

Studies in vitro have shown that substance P is degraded by angiotensin converting enzyme (‘ACE’: peptidyl dipeptidase A; EC 3.4.15.1; Skidgel et al., 1984), which forms part of the brain renin angiotensin system (BRAS). It is still not certain that ACE metabolises substance P in vivo (Mitchell et al., 2013) and, in any case, ACE is not the only peptidase that metabolises this peptide (Oblin et al., 1988). Nevertheless, a substantial body of evidence indicates that the BRAS regulates both locomotor activity and executive function (for recent review, see: Wright and Harding, 2011). For instance, ACE inhibitors improve performance in several preclinical screens of learning and memory, such as the Morris water maze and tests of active/passive avoidance (e.g., Barnes et al., 1992; Nikolova et al., 2000). ACE inhibitors also enhance cognitive performance in hypertensive patients and healthy controls, as well as in patients with dementia (Croog et al., 1986; Currie et al., 1990; Rozzini et al., 2006). Moreover, histochemical markers indicate that the BRAS is distributed across neuronal networks that have been strongly implicated in ADHD and motor control. For example, both ACE and angiotensin (AT) receptors are densely expressed within the basal ganglia, in regions such as the dorsal striatum, globus pallidus and substantia nigra (Strittmatter et al., 1984; Chai et al., 1987; Allen et al., 1992).

We reasoned that if ACE degrades substance P in vivo, then inhibition of this enzyme would reduce locomotor activity of wildtypes but would not affect NK1R−/− mice because they lack functional NK1R. Even if substance P fragments bind to and activate other sites, inhibition of ACE should modify the locomotor activity of wildtype and NK1R−/− mice in different ways. To test this possibility, we compared the locomotor activity of male NK1R−/− mice and their wildtypes in a light/dark exploration box (LDEB) following administration of the ACE inhibitor, captopril. Unlike many ACE inhibitors, this compound penetrates the brain in its active form (Geppetti et al., 1987; Ranadive et al., 1992). A caveat to this experiment was prompted by reports that ADHD, especially of the predominantly hyperactive/impulsive subtype, is more common in boys than girls (Waddell and McCarthy, 2012). There is also a report suggesting sex differences in ACE activity, which is reduced by oestrogen (Komukai et al., 2010). In light of this evidence, we compared the effects of captopril on the locomotor activity of both male and female NK1R−/− mice and their wildtype counterparts.

Contrary to our prediction, treatment with captopril reduced the locomotor activity of male NK1R−/− mice but did not affect that of male wildtypes, or female mice of either genotype. Given that ACE is better known for converting the (presumed) inactive precursor, angiotensin I, to the active product, angiotensin II (AngII), an obvious possibility is that this behavioural response to captopril could be due to a deficit in angiotensin II production. If so, this response should be mimicked by drug antagonism of AngII (type 1 (AT_1_) and/or type 2 (AT_2_)) receptors, which are expressed by neurones and glial cells in subcortical regions, including the striatum (Allen et al., 1992). To investigate this proposal, we compared the locomotor response of the two genotypes after treatment with either a selective AT_1_ receptor antagonist (losartan) or AT_2_ receptor antagonist (PD 123319).

Finally, there is extensive evidence that the BRAS modulates cognitive performance. For instance, several early studies suggested that captopril could have nootropic actions in rodents (e.g., Earley et al., 1989; Mondadori and Etienne, 1990; see: Wright and Harding, 2011). Against this background, a third experiment compared the effects of captopril on the cognitive performance and response control of male NK1R−/− and wildtype mice in the 5-CSRTT. We used this protocol because NK1R−/− mice have previously demonstrated both increased *premature responses* (an index of one form of impulsivity) and increased *omissions* (an index of inattentiveness) in this test (Dudley et al., 2013; Yan et al., 2011). Furthermore, it has been concluded, from a battery of studies measuring impulsivity in ADHD patients, that premature responses are “…the most sensitive measures for discriminating ADHD from control children” (Rubia et al., 2007). Consequently, the measurement of *premature responses* by NK1R−/− mice in the 5-CSRTT has strong translational relevance for ADHD research.

## 2 Experimental procedures

These experiments were authorised under the UK Animals (Scientific Procedures) Act 1986 and received approval from the local Animal Welfare and Ethical Review Body (UCL). This report complies with the ARRIVE guidelines for reporting of animal experiments.

### 2.1 Animals

All the animals were from inbred colonies maintained at University College London. NK1R−/− mice and their wildtype counterparts were of background strain, 129/Sv×C57BL/6J, crossed with an outbred MF1 strain many generations ago (described fully in: de Felipe et al. 1998). Animals were housed (2–5 mice per cage) in a holding room at 21±2 °C, 45±5% humidity, with a 12:12 h light:dark cycle (lighting increased in steps from 07.00 to 08.00 h and reduced in steps from 19.00 to 20.00 h). The home-cages incorporated environmental enrichment and sawdust bedding (3Rs Bedding Pty, Ltd) and were cleaned twice weekly. Water and food (2018 global Rodent Diet (Harlan)) were freely available for mice that were used to monitor locomotor activity, but mice destined for the 5-CSRTT were subjected to a restricted diet (see below).

### 2.2 Drugs

Captopril (*N*-[(*S*)-3-mercapto-2-methylpropionyl]-l-proline) was purchased from Sigma Aldrich, UK, losartan potassium (2-butyl-4-chloro-1-[[2′-(1*H*-tetrazol-5-yl)[1,1′-biphenyl]-4-yl]methyl]-1*H*-imidazole-5-methanol monopotassium salt) was purchased from LKT laboratories, UK, and PD 123319 ditrifluoroacetate (1-[[4-(dimethylamino)-3-methylphenyl]methyl]-5-(diphenylacetyl)-4,5,6,7-tetrahydro-1*H*-imidazo[4,5-*c*]pyridine-6-carboxylic acid ditrifluoroacetate) was purchased from Tocris, UK. All drugs were dissolved in 0.9% saline and administered by intraperitoneal (i.p.) injection in a volume of 10 mL/kg. The choice of doses was informed by published reports confirming a physiological or behavioural response to these drugs in mice and/or rats in vivo (e.g., captopril: Raghavendra et al., 2001; losartan: Vijayapandi and Nagappi, 2005; PD 123319: Macova et al., 2009).

### 2.3 Experimental design

#### 2.3.1 Experiment 1: Effects of captopril on locomotor activity of male and female NK1R−/− and wildtype mice

Male and female wildtype and NK1R−/− mice were used (i.e., four groups, *N*=6 for each group). They were 6–11 weeks of age and weighed 20–37 g. Animals were selected from a total of nine breeding pairs per genotype and selected to age-match the four groups as closely as possible.

Animals’ locomotor activity was monitored using a light/dark exploration box (LDEB; described fully in: Yan et al., 2010). Briefly, the mice were brought into the laboratory and weighed at 09.30 h and allowed to habituate to the test room for at least 3 h. At 13.00 h, they were placed into the dark zone of the LDEB and, after 60 min habituation, received a randomly assigned intraperitoneal (i.p.) injection of either captopril (10 or 25 mg/kg), or vehicle control (0.9% saline) or they were left untreated (no injection, ‘NI’). After a further 30 min (i.e., a total of 90 min in the dark zone), animals were transferred to the light zone of the LDEB and allowed free movement across both zones for 30 min. Their behaviour was recorded digitally and scored ‘blind’, later.

#### 2.3.2 Experiment 2: Effects of AT_1_ and AT_2_ receptor antagonism on locomotor activity of male NK1R−/− and wildtype mice

Because hyperactivity and a response to captopril were evident only in male NK1R−/− mice, female mice were not used in this experiment. When testing losartan, the male mice were 6–10 weeks of age (25–41 g; *N*=6 per group). A separate batch of mice was used to test the effects of PD 123319, and were 7-12 weeks of age (29-40 g; *N*=5 per group). In both cases, animals were selected from eight to nine different breeding pairs per genotype and selected so as to age-match the groups as closely as possible.

The protocol was similar to that used in experiment 1. One batch of animals received a randomly-assigned injection of either losartan (10 or 25 mg/kg, i.p.) or vehicle (0.9% saline, i.p.) following 30 min habituation to the dark zone of the LDEB. In a second batch of animals, losartan was replaced by PD 123319 (1 or 3 mg/kg, i.p.). All animals were transferred to the light zone 60 min after injection (i.e., a total of 90 min in the dark zone) and allowed free movement across both zones for 30 min. Their behaviour was recorded digitally and scored ‘blind’, later.

#### 2.3.3 Experiment 3: Effects of captopril on the performance of male NK1R−/− and wildtype mice in the 5-choice serial reaction-time task

Again, because a response to captopril was evident only in male NK1R−/− mice, this experiment did not study females. The males were aged 6–7 weeks and weighed 23–33 g at the start of testing (*N*=12 per group). Animals were selected from a total of three separate breeding pairs per genotype and were age-matched as closely as possible.

The protocol is described fully in Yan et al. (2011). All animals were subject to restricted food intake in order to stabilise their body weight at 90% of their free-feeding weight. Animals were brought to the laboratory between 09.00 and 09.30 h Monday to Friday, and trained/tested in the 5-CSRTT in either a morning session (10.00–12.00 h) or afternoon session (13.00–15.00 h). An equal number of wildtypes and NK1R−/− mice were balanced across the morning and afternoon sessions. Following habituation to the apparatus, animals were trained to nose-poke in response to a light stimulus that appeared in one of five equally spaced nose-poke apertures on one of the walls of the 5-CSRTT chamber. A *correct response* (nose-poking in the correct hole within the limited time-frame) triggered delivery of a sweetened milk reward (0.01 mL of 30% condensed milk solution) from a magazine in the opposite wall of the chamber. An *incorrect response* (nose-poking into a hole other than that signalled by the light stimulus), or an *omission* (failing to respond), or a *premature response* all resulted in a ’time-out’ punishment (i.e., the house-light of the apparatus was turned off and no reward was provided).

Animals were required to graduate through a series of six training stages before testing. The training stages were made progressively more difficult by: increasing the length of time before the light stimulus appears (intertrial interval, ‘ITI’); decreasing the length of time the animal has to respond to the light stimulus (limited hold, ‘LH’); decreasing the duration of the light stimulus (stimulus duration, ‘SD’). To achieve the baseline for testing, the animals were required to achieve specific performance criteria based on: the total number of trials completed; the number of correct responses completed; %accuracy; and %omissions (see: Yan et al., 2011).

Once the mice had attained stable baseline performance (100 completed trials, >50 correct trials, >75% accuracy, <25% omissions), untreated (uninjected) subjects were tested using two different test sessions: a long intertrial interval (LITI) test, which increases the ITI from 5 s to 10 s, and a variable intertrial interval (VITI) test, in which the ITI is variable (2, 5, 10 and 15 s) and delivered on a random schedule.

Animals experienced a series of once-weekly tests following either a second test with no injection (‘NI’), to serve as the baseline for the series of tests, a vehicle injection (0.9% saline, 10 mL/kg, i.p.), or captopril injection (5, 10 or 25 mg/kg, i.p.). All five treatments (NI, vehicle and captopril (3 doses)) were tested with both the VITI and LITI (10 testing sessions in total). Every animal experienced each test condition once, only, in a sequence that was counterbalanced (William’s Latin-square) across both experimental factors (genotype and time-of-day). All testing was carried out on Fridays, only. On intervening days, animals repeated Stage 6 of training, to ensure that stable baseline performance criteria were re-established before the next test.

### 2.4 Statistical analysis

The software package InVivoStat (Clark et al., 2012) was used for all statistical analyses, which were carried out on raw data unless diagnostic plots for normality of the data-set and equality of the variance of the samples suggested that transformation (square-root(score) or Log_10_(score+1)) was necessary.

In the LDEB experiments, data were analysed using a multifactorial 3-way single measures ANOVA with ‘genotype’, ‘treatment’ and ‘sex’ as between-subjects factors. In the 5-CSRTT experiment, data were analysed using a repeated measures (mixed model) ANOVA with ‘genotype’ and ‘time-of-day’ as the between-subjects factors and ‘treatment’ as the within-subjects factor. For both experiments, a significant effect of one of the main factors, or an interaction between them, was used as the criterion for progressing to post-hoc (LSD) comparisons.

## 3 Results

### 3.1 The hyperactivity of uninjected NK1R−/− mice is evident in male animals, only

**Fig. 1:**
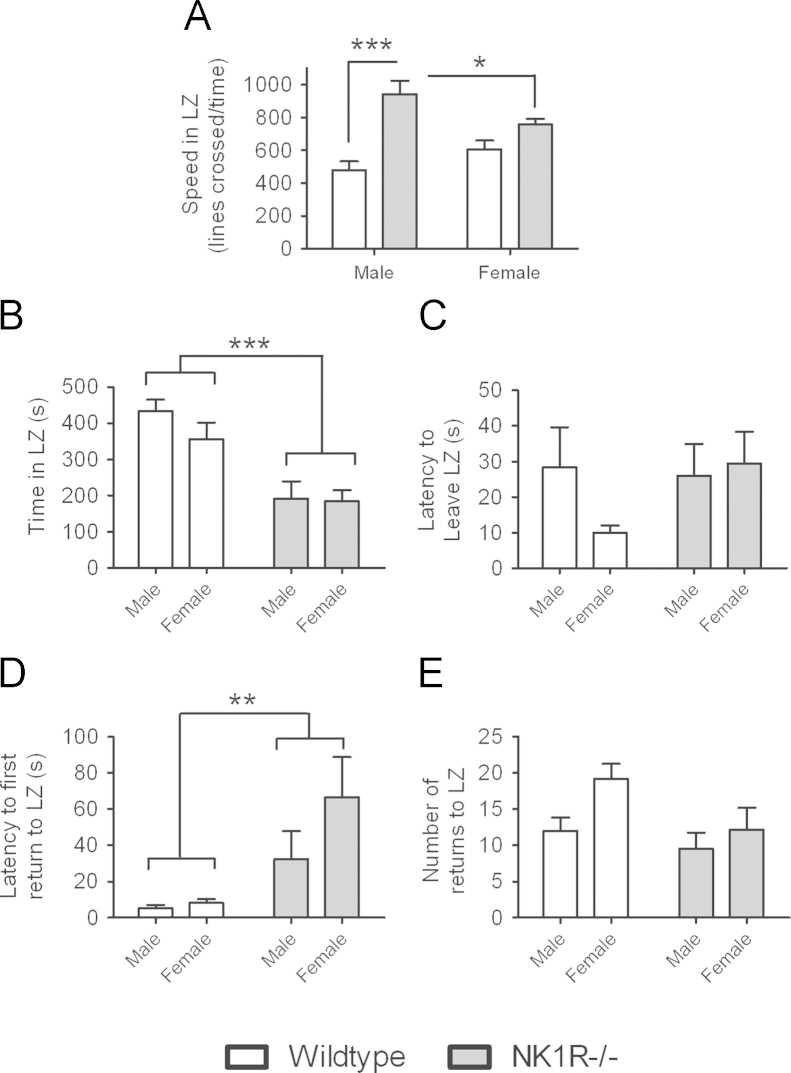
**Sex differences in the locomotor activity of (uninjected) wildtype and NK1R−/− mice in the light/dark exploration box.** Locomotor activity, measured as ‘Speed in Light Zone’ (LZ; lines crossed/time) (A). Time in LZ (B). Latency to leave LZ (C). Latency to first return to LZ (D). Number of returns to LZ (E). Bars indicate mean±s.e.m. Lines indicate statistically significant differences between groups. * *P*<0.05, ** *P*<0.01, *** *P*<0.001. *N*=6 per group. Data were analysed using single measures two-way ANOVA followed by post hoc (LSD) pair-wise comparisons.

In line with previous reports, the *locomotor activity* of male NK1R−/− mice was greater than that of male wildtype mice (LSD: *P*<0.001; Fig. 1A). We now further report that this hyperactivity of NK1R−/− mice depends on their sex (genotype×sex interaction: *F*(1,20)=7.41, *P*=0.013). Not only was the locomotor activity of male NK1R−/− mice greater than that of their female counterparts (LSD: *P*=0.033), but the locomotor activity of female mice did not differ in the two genotypes (LSD: *P*=0.074).

NK1R−/− mice spent less *time in the light zone* (main effect of genotype: *F*(1,22)=27.77, *P*<0.001; Fig. 1B) and took longer to *first return to the light zone* (an index of passive avoidance; main effect of genotype: *F*(1,22)=11.22, *P*=0.003; Fig. 1D) compared with wildtype mice. However, the two genotypes did not differ in either their *latency to leave the light zone* (an index of active avoidance; Fig. 1C) or the *number of returns to the light zone* (Fig. 1E). None of these behaviours differed in males and females (Fig. 1B–E).

### 3.2 The ACE inhibitor, captopril, reduces locomotor activity of male NK1R−/− mice, only

**Fig. 2:**
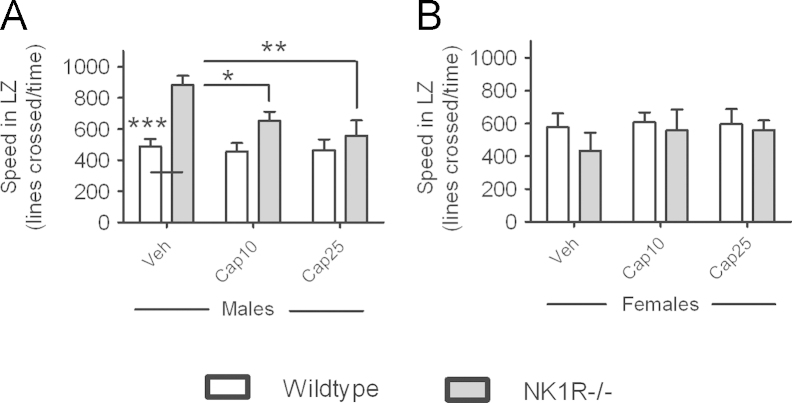
**The ACE inhibitor, captopril, reduced the locomotor activity of male NK1R−/− mice, only, in the light/dark exploration box.** Effects of captopril on locomotor activity, measured as ‘Speed in light zone’ (LZ; lines crossed/time), of male wildtype and NK1R−/− mice (A) and female wildtype and NK1R−/− mice (B). Bars indicate mean±s.e.m. Lines indicate statistically significant differences between groups. * *P*<0.05, ** *P*<0.01, *** *P*<0.001. *N*=6 per group. Data were analysed using single measures three-way ANOVA followed by post hoc (LSD) pair-wise comparisons. Veh, vehicle; Cap10, 10 mg/kg captopril; Cap25, 25 mg/kg captopril.

Both doses of captopril reduced the *locomotor activity* of male NK1R−/− mice, abolishing their hyperactivity (LSD (cf., vehicle): *P*=0.05 (10 mg/kg) and *P*=0.006 (25 mg/kg); Fig. 2A). However, neither dose affected the *locomotor activity* of either male wildtype mice (Fig. 2A) or females of either genotype (Fig. 2B). However, this interaction between sex and treatment just missed the criterion for significance (sex×treatment interaction: *F*(2,60)=2.73, *P*=0.073).

Captopril did not affect any of the ‘anxiety-like’ behaviours of either genotype, at either dose (Table 1).

### 3.3 AT_1_ receptor antagonism by losartan increases locomotor activity in NK1R−/− mice, only

**Fig. 3:**
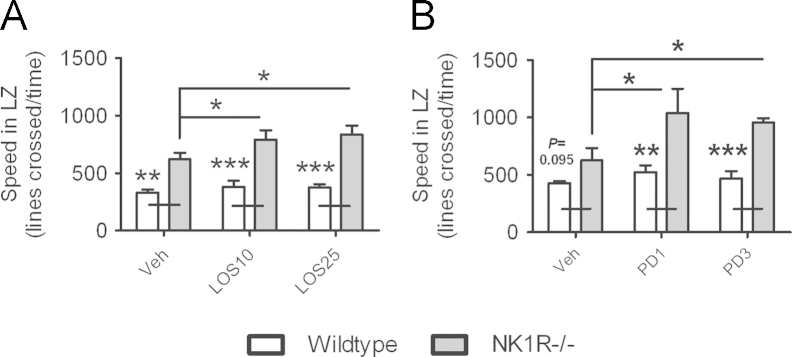
**The AT**_**1**_**receptor antagonist, losartan, and the AT**_**2**_**receptor antagonist, PD 123319, increased the locomotor activity of male NK1R−/− mice in the light/dark exploration box.** Effects of losartan on the locomotor activity, measured as ‘Speed in light zone’ (LZ; lines crossed/time), of male wildtype and NK1R−/− mice (A). Effects of PD 123319 on the locomotor activity, measured as ‘Speed in light zone (LZ)’ (lines crossed/time), of male wildtype and NK1R−/− mice (B). Bars indicate mean±s.e.m. Lines indicate statistically significant differences between groups. * *P*<0.05, ** *P*<0.01, *** *P*<0.001. *N*=5–6 per group. Data were analysed using single measures two-way ANOVA followed by post hoc (LSD) pair-wise comparisons. Veh, vehicle; Los10, 10 mg/kg losartan; Los25, 25 mg/kg losartan; PD1, 1 mg/kg PD 123319; PD3, 3 mg/kg PD 123319.

*Locomotor activity* was greater, overall, in NK1R−/− mice compared with wildtypes (main effect of genotype: *F*(1,30)=66.72; *P*<0.001). An overall effect of losartan on locomotor activity just missed the criterion for significance (main effect of treatment: *F*(2,30)=2.88; *P*=0.072). Post-hoc analyses revealed that, in contrast to captopril, both doses of losartan increased the locomotor activity of NK1R−/− mice (LSD (cf., vehicle): *P*=0.049 (10 mg/kg) and *P*=0.015 (25 mg/kg); Fig. 3A) but did not affect that of wildtype mice.

Losartan did not affect any of the ‘anxiety-like’ behaviours of either genotype, at either dose (Table 1).

### 3.4 AT_2_ receptor antagonism by PD 123319 does not affect locomotor activity of NK1R−/− or wildtype mice

Again, *locomotor activity* was greater, overall, in NK1R−/− mice compared with wildtypes (main effect of genotype: *F*(1,24)=29.51; *P*<0.001). There was also an overall effect of PD 123319 on locomotor activity (main effect of treatment: *F*(2,24)=3.99; *P*=0.032). Post-hoc analyses revealed that, as with losartan, both doses of PD 123319 increased the locomotor activity of NK1R−/− mice (LSD (cf. vehicle): *P*=0.01 (1 mg/kg) and *P*=0.013 (3 mg/kg); Fig. 3B) but did not affect that of wildtypes.

PD 123319 did not affect any of the ‘anxiety-like’ behaviours of either genotype, at either dose (Table 1).

### 3.5 The effects of the ACE inhibitor, captopril, on the incidence of *premature responses* and *%omissions* in the 5-CSRTT

All mice achieved the stable baseline (Stage 6 of training) performance criteria for testing in the 5-CSRTT.

**Fig. 4:**
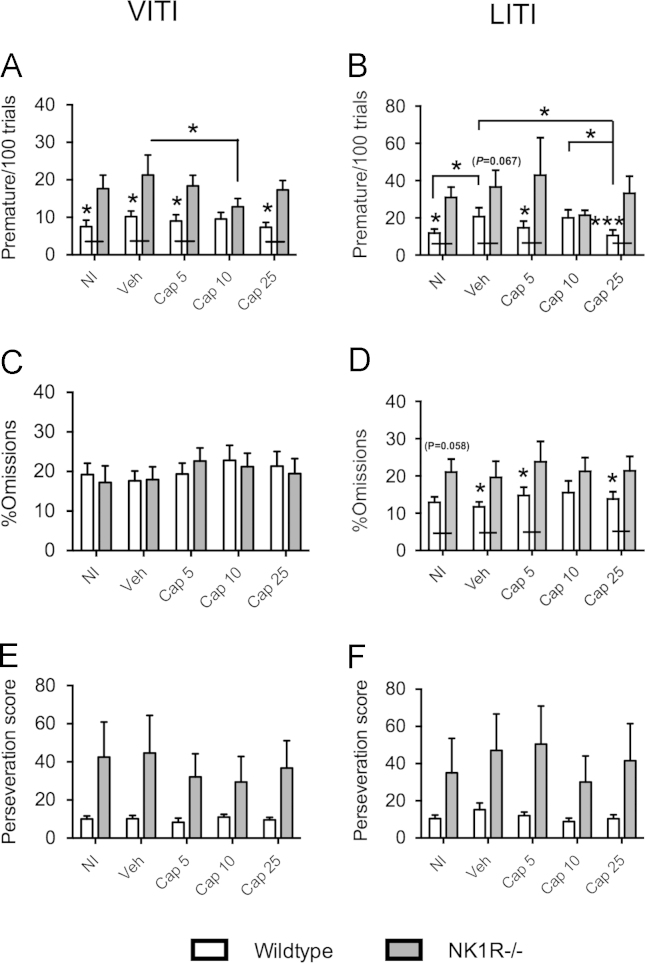
**The angiotensin converting enzyme inhibitor, captopril, improved the performance (impulsivity and inattentiveness) of NK1R−/− mice in the 5-choice serial reaction-time task.** Effects of captopril on: *premature responses* (*per 100 trials*) in the VITI (A) and LITI (B); *%omissions* in the VITI (C) and LITI (D); *perseveration* in the VITI (E) and LITI (F). Bars indicate mean±s.e.m. Lines indicate statistically significant differences between groups. * *P*<0.05, ** *P*<0.01, *** *P*<0.001. *N*=9–12 per group. Data were analysed using mixed model three-way ANOVA followed by post hoc (LSD) pair-wise comparisons. NI, no injection; Veh, vehicle; Cap5, 5 mg/kg captopril; Cap10, 10 mg/kg captopril; Cap25, 25 mg/kg captopril.

In both the VITI and the LITI, *premature responses* (*per 100 trials*) were greater, overall, in NK1R−/− mice compared with wildtype mice (VITI: *F*(1,22)=18.77, *P*<0.001; LITI: *F*(1,22)=10.97, *P*=0.003; Fig. 4A and B). In the VITI, treatment with captopril (10 mg/kg) reduced the incidence of *premature responses* in NK1R−/− mice, only (LSD (cf., vehicle): *P*=0.033). In the LITI, treatment with this dose of captopril (10 mg/kg) similarly abolished the genotype difference in *premature responses*, whilst treatment with the higher dose (25 mg/kg) reduced the incidence of this behaviour in wildtypes (LSD (cf., vehicle): *P*=0.012; LSD (cf., 10 mg/kg): *P*=0.02) but not NK1R−/− mice.

In the VITI, there were no genotype differences in *%omissions*, and no effects of captopril, at any dose (Fig. 4C). However, in the LITI, *%omissions* were higher, overall, in NK1R−/− mice compared with wildtypes (main effect of genotype: *F*(1,22)=5.8, *P*=0.025; Fig. 4D). This genotype difference was abolished in mice given the intermediate dose of captopril (10 mg/kg).

There were no genotype differences in *perseveration* in either the VITI or the LITI, and captopril did not affect this behaviour, in either test (Fig. 4E and F).

### 3.6 Other behaviours in the 5-CSRTT

**Fig. 5:**
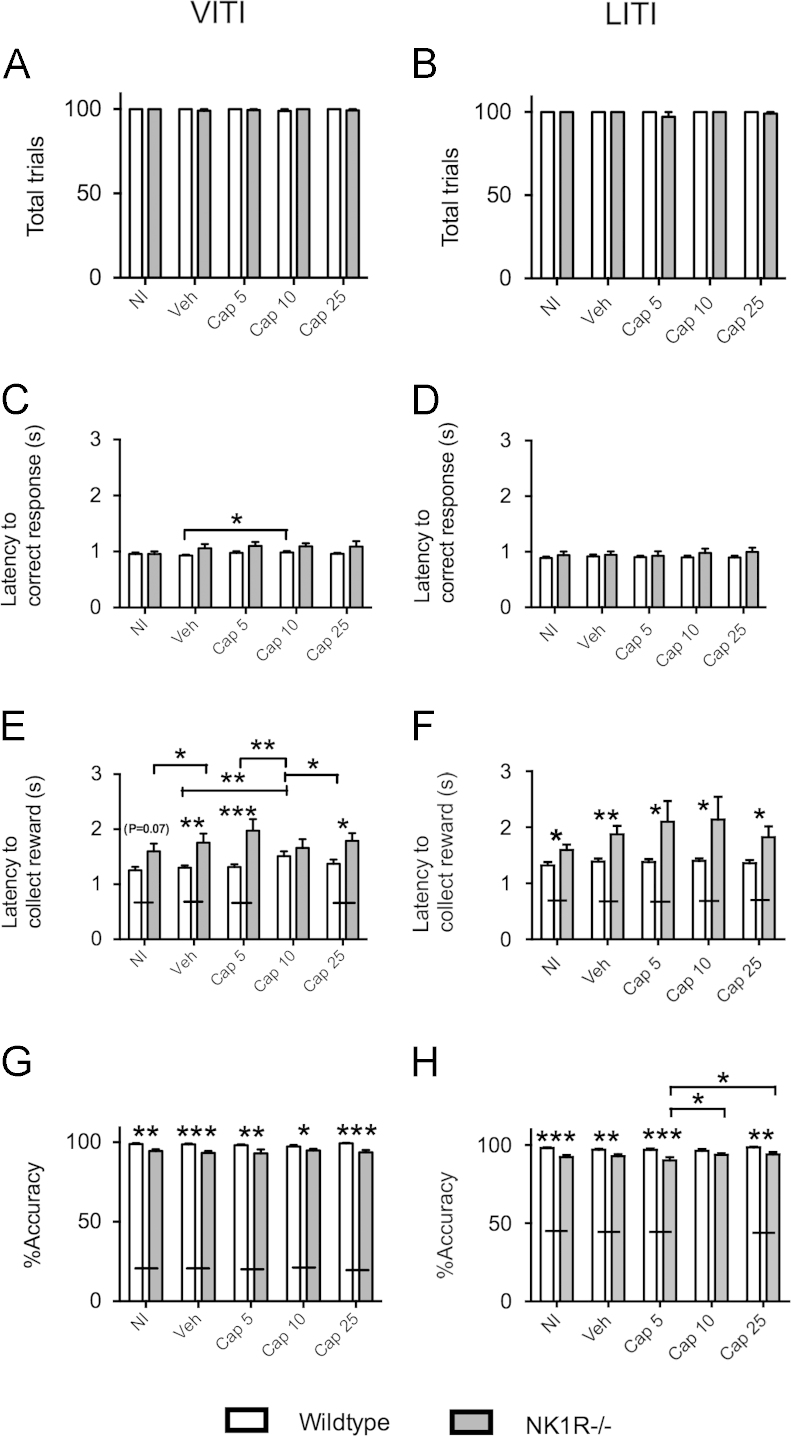
**The effects of the angiotensin converting enzyme inhibitor, captopril, on the performance of wildtype and NK1R−/− mice in the 5-choice serial reaction-time task: total trials, accuracy and latencies.** Effects of captopril on: *total trials* in the VITI (A) and LITI (B); *latency to correct response* in the VITI (C) and LITI (D); *latency to collect reward* in the VITI (E) and LITI (F); *%accuracy* in the VITI (G) and LITI (H). Bars indicate mean±s.e.m. Lines indicate statistically significant differences between groups. * *P*<0.05, ** *P*<0.01, *** *P*<0.001. *N*=9–12 per group. Data were analysed using mixed model three-way ANOVA followed by post hoc (LSD) pair-wise comparisons. NI, no injection; Veh, vehicle; Cap5, 5 mg/kg captopril; Cap10, 10 mg/kg captopril; Cap25, 25 mg/kg captopril.

There was no genotype difference in *total trials* in either the VITI or the LITI, and this behaviour was unaffected by captopril (Fig. 5A and B). *Latency to correct response* also did not differ in the two genotypes in either test but, in the VITI, captopril (10 mg/kg) slightly (c. 0.05 s) increased this latency to respond in wildtype mice, only (*F*(4,72)=2.72, *P*=0.036; LSD: *P*=0.042; Fig. 5C and D). *Latency to collect the reward* was greater in NK1R−/− mice in both tests (VITI: *F*(1,21)=8.24, *P*=0.009; LITI: *F*(1,21)=8.32, *P*=0.009; Fig. 5E and F). However, in the VITI, captopril (10 mg/kg) increased the latencies of wildtypes and abolished the genotype difference in this measure (genotype×treatment interaction: *F*(4,69)=3.13, *P*=0.02). *%Accuracy* in both tests was slightly lower, overall, in NK1R−/− mice (VITI: *F*(1,22)=14.75, *P*<0.001; LITI: *F*(1,22)=22.08, *P*<0.001) (Fig. 5G and H). Captopril did not affect *%accuracy* in the VITI test but, in the LITI test, the lowest dose (5 mg/kg) slightly reduced this index of attention (c. 3%) in NK1R−/− mice.

## 4 Discussion

We have investigated further the behavioural abnormalities of inbred NK1R−/− mice.

### 4.1 Sex differences in the locomotor activity of NK1R−/− mice

The first aim of this study was to establish whether the locomotor hyperactivity of male NK1R−/− mice, which we have reported previously (Fisher et al., 2007; Herpfer et al., 2005; Yan et al., 2010), is also evident in female NK1R−/− mice. Whereas male NK1R−/− mice were hyperactive compared with their wildtype counterparts, the locomotor activity of female NK1R−/− mice did not differ from wildtypes.

This finding is potentially important because there are clear differences in the symptom profile of male and female ADHD patients: males more commonly present as the predominantly hyperactive/impulsive or combined subtype, whereas females typically express the predominantly inattentive subtype (Staller and Faraone, 2006). Males with ADHD also have a higher incidence of other externalised disruptive behaviours (Rucklidge, 2010) and are more susceptible to disorders of excitability and movement, such as Tourette’s syndrome or Parkinson’s disease (Haaxma et al., 2007; Shulman, 2007). The possibility that impaired *TACR1* function contributes to these abnormalities merits further investigation.

Motor function is regulated by dopaminergic corticostriatal circuitry, and extensive evidence points to disruption of these networks in ADHD (see: del Campo et al., 2011). These circuits are modulated by gonadal steroids, during both perinatal development and puberty, and display sexual differentiation in male and female brains (Waddell and McCarthy, 2012). For instance, sex differences in the distribution and density of dopamine D1 and D2 receptors have been observed in the nucleus accumbens, dorsal striatum and prefrontal cortex of juvenile rats, with females experiencing smaller increases in receptor expression during puberty than males (Anderson and Teicher, 2000). It is argued that over-expression of dopamine receptors, when coupled with pre-existing abnormalities in monoaminergic transmission observed in patients with ADHD, could contribute to the greater hyperactivity observed in male patients, compared with females (Waddell and McCarthy, 2012). There is extensive evidence to suggest that a lack of functional NK1R also disrupts dopaminergic transmission in corticostriatal regions (reviewed by: Stanford, 2014). Findings reported here suggest that a functional deficit in NK1R, and, by inference, polymorphisms of the *TACR1* gene, could contribute to sex differences in expression of hyperactivity in ADHD.

### 4.2 Angiotensin converting enzyme and the regulation of motor function

The second aim of this study was to determine the effects of the angiotensin converting enzyme (ACE) inhibitor, captopril, on the locomotor activity of NK1R−/− mice. This was prompted by evidence that substance P, the preferred ligand for NK1R, is metabolised by ACE (Skidgel et al., 1984). On this basis, it was predicted that captopril, by increasing the availability of substance P, would reduce the locomotor activity of wildtype mice but leave NK1R−/− mice unaffected. Contrary to this prediction, captopril prevented the hyperactivity of male NK1R−/− mice without affecting the locomotor activity of wildtypes. This genotype-specific response to captopril suggests that locomotor hyperactivity, caused by a lack of functional NK1R in male mice, can be prevented by reducing ACE activity. This effect cannot be explained by increased activation of NK1R by substance P because this peptide would be ineffective in NK1R−/− mice. Nonetheless, there are several alternative explanations for this response to captopril. The most obvious of these is the prevention of angiotensin II (AngII) production, which is formed from the hydrolysis of angiotensin I (AngI) by ACE. Although published findings are somewhat inconsistent, there are several reports that intracerebroventricular administration of AngII increases locomotor activity (see: Braszko, 2002 and references therein), which is in line with the reduction in hyperactivity in captopril-treated mice found here.

A further interesting finding was that captopril influenced the locomotor activity of male, but not female, NK1R−/− mice. The inference that there are sex differences in the influence of ACE on motor function is in line with evidence that gonadal steroids influence the activity of ACE, which is increased by testosterone and reduced by oestrogen (Komukai et al., 2010). Indeed, low baseline levels of ACE could explain why, in our experiment, captopril was ineffective in females. A difference in baseline activity of ACE in male and female animals could also have implications for sex differences in hyperactivity in ADHD, as well as its potential amelioration by captopril.

To test whether or not the effect of captopril in male NK1R−/− mice was due to a reduction in AngII, we went on to investigate the effects of the AT_1_ receptor antagonist, losartan, and the AT_2_ receptor antagonist, PD 123319, on the locomotor activity of the two genotypes. We reasoned that, if the prevention of hyperactivity of NK1R−/− mice by captopril is due to a reduction in AngII availability, then that response to captopril should be mimicked by antagonism of AT_1_ receptors and/or AT_2_ receptors. In fact, both AngII receptor antagonists *increased*, rather than reduced, the locomotor activity of NK1R−/− mice, but neither compound affected wildtype mice.

Previous reports concerning the effects of AngII receptor antagonism on locomotor activity are somewhat inconsistent. In studies of male outbred Wistar rats, losartan and PD 123319 were both ineffective when administered alone (Braszko, 2002). Losartan was also ineffective in the spontaneously hypertensive rat, the most extensively studied rodent model of ADHD (Irvine et al., 1995). Although these results are consistent with the lack of effect of either antagonist in wildtypes seen here, in another study, losartan decreased locomotor activity in outbred BALB/c mice (Raghavendra et al., 1998). Both losartan and PD 123319 also abolished an increase in locomotor activity following intracerebroventricular administration of AngII in rats (Braszko, 2002).

All of these findings stand in contrast to the increase in locomotor activity of NK1R−/− mice, following administration of losartan and PD 123319, in this study. Nevertheless, our findings suggest that the influence of angiotensin receptors on motor function is disrupted in these mice, which again points to a functional interaction between the BRAS and NK1R in the regulation of motor activity.

The proposal that both ACE and AngII receptors are involved in the regulation of motor function is supported by their high densities in both the striatum and the substantia nigra, albeit with different distributions. Specifically, angiotensin receptors are found on nigrostriatal dopaminergic nerve terminals (Allen et al., 1992), whereas the density of ACE is highest on striatonigral and striatopallidal neurons (Chai et al., 1987). There is also evidence that ACE inhibition increases striatal dopamine efflux (Jenkins, 2008), which could underlie its influence on locomotor activity. This is an interesting possibility because captopril increases the striatal concentration of the substance P metabolic fragment, substance P(1–7) (Michael-Titus et al., 2002), which increases striatal dopamine release in a concentration-dependent manner (Reid et al., 1990). Importantly, this dopamine response is mediated by an NK1R-independent mechanism and so could occur in NK1R−/− mice. Nevertheless, several other neuropeptides (e.g., bradykinin, neurotensin, dynorphin and enkephalin), are also metabolised by ACE (Skidgel et al., 1984). It cannot be ruled out that changes in the availability of any, or all, of these peptides contribute to the behavioural response to captopril.

Whatever the mechanism, it is clear that any change in locomotor activity triggered by either ACE inhibitors, or AngII receptor antagonists, is prevented by functional NK1R. This finding could be exploited therapeutically because it suggests that ACE inhibition could be beneficial in reducing the hyperactivity of (male) ADHD patients with polymorphism of the *TACR1* gene.

### 4.3 Angiotensin converting enzyme and impulsivity/inattention in the 5-CSRTT

The third aim of this study was to determine whether captopril would improve the cognitive performance of NK1R−/− mice in the 5-CSRTT. This seems to be the case because captopril dose-dependently abolished the genotype difference in *premature responses* in both the VITI and the LITI test, as well as the genotype difference in *omissions* in the LITI test. Neither of these responses is likely to be explained by any change in motivation to carry out the task because, in NK1R−/− mice, captopril did not reduce *total trials* or increase either the *latency to correct response* or *latency to collect the reward*. We also do not believe that the lack of any change in *%omissions* in the VITI is due to a floor effect because, in a previous study using this test, treatment with guanfacine reduced this measure in NK1R−/− mice, only (Pillidge et al., 2014).

There are many reports that ACE inhibitors improve cognitive performance (for review, see: Wright and Harding, 2011) but this is the first instance of such a drug being tested in the 5-CSRTT. The mechanisms underlying this effect are, as yet, unknown. However, as with motor activity, the response to captopril in this test cannot be explained by an increase in substance P transmission. Instead, functional changes in any of a range of neuropeptides could contribute to the response to captopril in NK1R−/− mice (see above). Another possibility, not explored here, is that the improved cognitive performance is mediated by the AngII fragment angiotensin IV, which activates angiotensin type 4 (AT_4_) receptors (see: Wright and Harding, 2011). It is also striking that the improvements in *premature responses* seen in NK1R−/− mice treated with captopril showed a bell-shaped dose-dependent response. This bears similarities to that of other drugs that are used to treat ADHD, such as methylphenidate, which can show beneficial effects at low but not higher doses (Tannock et al., 1995).

Impulsivity is widely believed to be associated with abnormal serotonergic transmission, dopaminergic transmission, or both (Oades, 2002), whereas impaired attention points more specifically to disruption of noradrenergic transmission (Aston-Jones and Cohen, 2005). Although there are limited reports directly investigating the effects of captopril on the function of these neurotransmitters, there is considerable indirect evidence to suggest that ACE inhibition would affect all three of these systems: neuropeptides that are metabolised by ACE affect the function of serotonin (Jenkins, 2008), dopamine (Prus et al., 2007) and noradrenaline (Sumners and Phillips, 1983) in neuronal networks implicated in ADHD. To the best of our knowledge, the results of this study offer the first evidence that the BRAS influences premature responses and that this abnormal behaviour is diminished by captopril, and possibly other ACE inhibitors. The effects of ACE inhibitors on the function of neuronal networks that influence this form of impulsivity warrant further investigation, especially in ADHD patients.

### 4.4 Conclusions

Here, we report that the hyperactivity of male NK1R−/− mice is not evident in female mice, revealing striking parallels between this abnormal behaviour and differences in the typical symptom profile of male and female patients with ADHD. We also report that the ACE inhibitor, captopril, prevents the hyperactivity, impulsivity and, possibly, inattentiveness, of male NK1R−/− mice. These findings point to a functional interaction between the BRAS and NK1R on these behaviours that merits further investigation. Our findings further suggest that captopril (and possibly other ACE inhibitors) could provide a novel therapeutic target for the treatment of ADHD, particularly in males expressing the Predominantly Hyperactive/Impulsive Subtype of this disorder.

## Declaration of originality & responsibility

These studies were funded by the Medical Research Council (UK). The sponsors had no role in the design, implementation, analysis, or interpretation of the data, in the preparation of the manuscript, or in the decision to submit the paper for publication. None of the material in this manuscript has been submitted for publication elsewhere.

## Contributors

Experimental design: AJP, SCS. 5-CSRTT (training): AJP, KP. 5-CSRTT (testing): AJP. LDEB: AJP, EG. Data analysis: AJP. Wrote the manuscript: AJP, SCS.

## Conflicts of interest

None of the authors has any conflict of interest to declare (SCS is a named inventor on an EU patent for the NK1R−/− mouse model of ADHD, but declined the option to receive royalties).

## Figures and Tables

**Table 1:** **Captopril, losartan and PD 123319 did not affect any of the ‘anxiety-like’ behaviours in the two genotypes when tested in the light/dark exploration box.** Values indicate overall effect of treatment (drug). LZ, light zone.

**Behaviour**	**Captopril**	**Losartan**	**PD 123319**
*Time in LZ (s)*	*F*(2,60)=0.41; *P*=0.666	*F*(2,30)=1.36; *P*=0.271	*F*(2,24)=0.84; *P*=0.445
*Latency to leave LZ (s)*	*F*(2,60)=0.5; *P*=0.609	*F*(2,30)=0.43; *P*=0.656	*F*(2,24)=0.45; *P*=0.645
*Latency to first return to LZ (s)*	*F*(2,60)=0.22; *P*=0.803	*F*(2,30)=0.16; *P*=0.856	*F*(2,22)=1.92; *P*=0.17
*Number of returns to LZ (s)*	*F*(2,60)=1.96; *P*=0.149	*F*(2,30)=1.35; *P*=0.274	*F*(2,24)=0.08; *P*=0.924
